# Alternative Lengthening of Telomeres in Pediatric High-Grade Glioma and Therapeutic Implications

**DOI:** 10.3390/cancers15123070

**Published:** 2023-06-06

**Authors:** Banlanjo Umaru, Satarupa Sengupta, Shiva Senthil Kumar, Rachid Drissi

**Affiliations:** 1 Center for Childhood Cancer Research, Nationwide Children’s Hospital, Columbus, OH 43205, USA; banlanjo.umaru@nationwidechildrens.org (B.U.); shiva.senthilkumar@nationwidechildrens.org (S.S.K.); 2 Division of Pulmonary, Critical Care, and Sleep Medicine, Department of Internal Medicine, University of Cincinnati College of Medicine, Cincinnati, OH 45267, USA; sengupsp@ucmail.uc.edu; 3 Department of Pediatrics, The Ohio State University College of Medicine, Columbus, OH 43210, USA

**Keywords:** telomeres, telomerase, alternative lengthening of telomeres, pediatric high-grade glioma

## Abstract

**Simple Summary:**

Pediatric high-grade gliomas (pHGGs) are highly aggressive tumors with a dismal prognosis despite multimodal therapy including surgery, radiation therapy and chemotherapy, underscoring the urgent need to develop novel therapeutic strategies. During tumor development, cells achieve immortality by activating telomere maintenance mechanisms. Alternative lengthening of telomere (ALT) is an important mechanism for maintaining telomere length and cell proliferation in tumor cells. However, the molecular pathway and prognostic significance of ALT activation in pHGGs are poorly understood. Here, we report the heterogeneity of telomere maintenance mechanisms and their association with genetic alterations with the presence of both ALT and telomerase activation in some tumors. These findings are particularly important for the future development of novel therapeutic strategies targeting ALT and telomerase in pHGGs.

**Abstract:**

Pediatric high-grade gliomas (pHGGs), including diffuse intrinsic pontine glioma (DIPG), are highly aggressive tumors with dismal prognoses despite multimodal therapy including surgery, radiation therapy, and chemotherapy. To achieve cellular immortality cancer cells must overcome replicative senescence and apoptosis by activating telomere maintenance mechanisms (TMMs) through the reactivation of telomerase activity or using alternative lengthening of telomere (ALT) pathways. Although the ALT phenotype is more prevalent in pHGGs compared to adult HGGs, the molecular pathway and the prognostic significance of ALT activation are not well understood in pHGGs. Here, we report the heterogeneity of TMM in pHGGs and their association with genetic alterations. Additionally, we show that sensitivity to the protein kinase ataxia telangiectasia- and RAD3-related protein (ATR) inhibitor and the ATR downstream target CHK1 is not specific to pHGG ALT-positive cells. Together, these findings underscore the need for novel therapeutic strategies to target ALT in pHGG tumors.

## 1. Introduction

Pediatric high-grade gliomas (pHGGs) are highly aggressive tumors with a dismal prognosis. The median overall survival (OS) is of <1 year for diffuse intrinsic pontine glioma (DIPG) and 5-year OS is of <10% for other pHGGs despite multimodal therapy [1,2,3,4,5], underscoring the urgent need to develop novel therapeutic strategies to improve outcomes.

During tumorigenesis, tumor cells overcome the barrier of replicative senescence and apoptosis by activating telomere maintenance mechanisms (TMMs) critical for genomic stability and replicative immortality. The well-known telomere-length maintenance mechanism involves the reactivation of the enzyme telomerase. The enzyme consists of two essential subunits: an RNA component hTERC in humans, and the catalytic protein subunit hTERT, which is the primary determinant of enzyme activity. Telomerase activity is present in 85–95% of human cancers; however, this activity is undetectable in most normal human somatic cells and tissues, including the brain [6,7]. Two *hTERT* promoter mutations (C228T and C250T) inducing telomerase activity have been identified in various cancers, including primary glioblastoma multiforme (GBM) with a higher percentage in adult GBM (58–84%) compared to pediatric GBM (3–11%) [8,9,10,11]. Higher levels of telomerase activity in adult GBM are associated with shorter overall survival (OS) [12,13,14].

Alternatively, some tumors use the telomere homologous recombination-mediated mechanism, called alternative lengthening of telomeres (ALT), particularly prevalent in tumors of mesenchymal and neuroepithelial origin, including brain tumors and soft-tissue sarcomas [15,16]. The ALT pathway is characterized at the cellular level by the presence of ALT-associated promyelocytic leukemia protein nuclear bodies (APBs), the presence of extrachromosomal telomeric DNA repeats in the form of C-Circles, high levels of telomere-sister chromatid exchanges, and a high frequency of heterogeneous telomere length and chromosomal instability [17,18,19,20]. The prevalence of ALT is variable among different tumor types with high frequency in tumors of the central nervous system (CNS), especially gliomas. In GBM, ALT is more prevalent in pHGG (44–53%) compared to adult HGG (11%) [15,21,22,23,24,25]. ALT-positive adult GBM were reported to have longer survival times [23,25], however, no significant association between ALT status and OS has been observed in pHGG, probably due to small sample sizes and typical short survival times of pHGG patients, especially in H3.3K27M mutant pHGGs [21,26].

Previous studies have demonstrated potential connections between *ATRX*/*DAXX, H3-3A* (encoding for H3.3 histone variant) mutations and ALT use, especially in oligodendrogliomas, medulloblastomas, neuroblastomas and GBM [27,28,29]. However, the presence of ALT phenotypes in pHGG, adult pancreatic neuroendocrine tumors and melanoma in the absence of *ATRX* mutations has recently been reported [26,30], suggesting the acquisition of these alterations is not necessary for the induction of ALT. Further investigations are needed to elucidate the mechanistic biology of ALT in these tumors.

Although mutations in *ATRX* and the *TERT* promoter are mutually exclusive, the coexistence of ALT and telomerase activity has been observed in some tumors, including neuroblastoma [31], sarcoma [32], breast cancers [33] and pHGG [21]. Despite these observations, the clinical implications are still elusive, especially for the coexistence of both TMMs in pHGG.

We report here the TMMs in pHGG, including DIPG along with the genetic alterations related to the ALT pathway and the sensitivity of ALT pHGG cells to ATR and CHK1 inhibition.

## 2. Materials and Methods

### 2.1. Tumor Tissue Samples, Cell Culture and Reagents

Tumor tissue samples with their matched normal tissue were obtained from the International DIPG/DMG Registry as described previously [34]. Briefly, patients with DIPG were informed and consented to the IRB-approved protocol (Study ID: 2016-3357) at Cincinnati Children’s Hospital Medical Center. Diagnosis of DIPG was based on clinical symptoms and imaging characteristics on pretreatment magnetic resonance imaging (MRI). Primary normal human foreskin fibroblasts (HFF) strains (ATCC CRL-2091), human cervical carcinoma cell lines (HeLa), and the human osteosarcoma cell lines (Saos-2) (ATCC HTB-85) were purchased from ATCC. HFF and HeLa cells were cultured in DMEM (Gibco) supplemented with 10% FBS, while Saos-2 cells were cultured in McCoy’s 5A medium supplemented with 15% FBS. Pediatric GBM cell lines, CCHMC-DIPG-1, R0315-GBM, SJ-HGGX42 and HSJD-GBM002, were cultured in neurosphere stem cell media as described elsewhere [35,36]. KNS42 cells were cultured in DMEM (Gibco) supplemented with 10% FBS. KNS42 cells were obtained from the JCRB (Japan Cancer Research Resources) cell bank. HSJD-GBM002 cells were generous gifts from Dr. Angel Montero Carcaboso. SJ-HGGX42 cells were obtained from PBTP (https://pbtp.stjude.cloud). ATR inhibitor VE-822 (S7102) and CHK1 inhibitor (Chir-124) (S2683) were purchased from Selleckchem.

### 2.2. Proliferation Assay

Cell proliferation was measured using WST-1 assay (Takara Bio Inc., Shiga, Japan ) as per the manufacturer’s instructions. WST-1 reagent was added to each well at a final dilution of 1:10, incubated for 1 h at 37 °C, and absorbance was measured at 450 nm with 650 nm as the reference wavelength.

### 2.3. Western Blotting

Immunoblot assays were performed as previously described [35]. Antibodies used were against ATR (Cell Signaling Technology, Danvers, MA, USA), ATR-T1989-P (GeneTex, Irvine, CA, USA), CHK1, CHK1-S345-P (ATR phosphorylation site), CHK1-S296-P (autophosphorylation site), β-Actin, H2AX S139-P, and H3.3S31-P (Cell Signaling Technology, Danvers, MA, USA). Bands visualized with ECL were captured using the ChemiDoc MP imaging system (Bio-Rad Laboratories, Hercules, CA, USA) and quantified using ImageJ software (National Institutes of Health, Bethesda, MD, USA).

### 2.4. Rolling C-Circle Amplification

DNA was extracted from tumor specimens using the Gentra Puregene kit (Qiagen, Hilden, Germany). Assessment of C-Circle formation was performed based on a previously described method [19]. In brief, following φ29 amplification, DNA was transferred to a charged nylon membrane and detection was carried out using the TeloTAGGG kit (Roche Diagnostics, Indianapolis, IN, USA) following the manufacturer’s instructions for telomere detection. DNA from Saos2 and HeLa cells were used as positive and negative controls, respectively. Samples showing a positive signal were considered to be ALT positive, while those with no signal were ALT negative.

### 2.5. TRAP Assay

Protein extraction and TRAP assay were performed as previously described [37]. A total of 25–100 ng of protein was used to perform the TRAP assay using the TRAPeze Telomerase Detection Kit (Millipore, Burlington, MA, USA) according to the manufacturer’s protocol.

### 2.6. hTERT Expression

RNA extraction, cDNA synthesis and qRT-PCR analysis were performed as previously described [21]. Primer/probe sets for *hTERT* (Hs00972650_m1) and *GAPDH* (Hs03929097_g1) were purchased from Applied Biosystems. Two independent qRT-PCR experiments were performed with each sample run in triplicates. Changes in gene expression (expressed as RQ) were calculated using the ΔΔCt method.

### 2.7. IF-FISH Assay (Immunofluorescence—Fluorescence In Situ Hybridization)

FFPE tissue sections were first deparaffinized. Then, heat-induced antigen retrieval (HIER) was performed using 10 mM citrate buffer (pH 6) with 0.05% tween-20 in a steamer for 15–20 min, followed by cooling and dehydration with a graded ethanol concentration series. After air-drying, the tissue sections were covered with a hybridization solution (70% formamide, 0.5% Blocking Reagent (Roche Diagnostics, Indianapolis, IN, USA) diluted in 100 mM maleic acid and 150 mM NaCl, and 10 mM Tris (pH 7.5) with 300 ng/mL telomere probe (PNA(CCCTAA)_3_-Cy3) (Biosynthesis, Lewisville, TX, USA), and denatured for 6 min at 84 °C and hybridized for 3–4 h at room temperature in the dark. Tissue sections were then washed twice with 70% formamide and 10 mM Tris (pH 7.5) and three times with PBS + 0.08% tween-20. After that, slides were incubated in a blocking solution (5% donkey serum, 0.3% Triton X-100 in 1X TBS) for 30 min, and treated with anti-ATRX (rabbit 1:250; Sigma, St. Louis, MO, USA) primary antibody for overnight at 4 °C. The next day, slides were washed (3 times in 1X TBST) and incubated with secondary antibodies (Alexa-Fluor 488 Conjugated Donkey anti-Rabbit 1:400 (Jackson ImmunoResearch, West Grove, PA, USA) for 1 h at room temperature. Finally, the tissue sections were washed, and embedded with mounting media with DAPI (Vector Laboratories H1200, Burlingame, CA, USA). Images were captured with a 60× oil objective in a confocal microscope (Nikon, Melville, NY, USA).

### 2.8. Immunofluorescence

Immunostaining was performed as described previously [38]. Primary antibodies were used against H3 S10-P (1:500, Cell Signaling Technology, Danvers, MA, USA), or H3.3 S31-P (1:500, Cell Signaling Technology, Danvers, MA, USA) with corresponding secondary antibodies (Alexa-Fluor 568– or 647–conjugated donkey anti-rabbit, or anti-mouse (1:500, Jackson ImmunoResearch, West Grove, PA, USA) as applicable. For nuclear staining, cells were embedded with mounting media with DAPI (Vector Laboratories H1200). Images were captured with a 60× oil objective on a Nikon Eclipse Ti2-E AXR confocal microscope.

### 2.9. H3-3A Gene Sequencing

DNA was extracted from tumor samples (RT-1969) and pHGG cell lines (R0315-GBM, HSJD-GBM002, SJ-HGGX42 and KNS42) using the Gentra Puregene kit (Qiagen, Hilden, Germany) following manufacturer’s instructions. Histone 3.3 and H3.1 genes (*H3-3A* and *H3C2*) were sequenced as previously described [21].

### 2.10. Whole Genome Sequencing

The whole genome sequencing was performed on DIPG tumor tissue along with matched normal brain tissue using the Illumina HiSeq 2500, BWA-mem on GRCh37 alignment and Mutect variant calling.

### 2.11. Orthotopic Implantation

The patient tumor sample received through consent was immediately dissociated and prepared for injection. To reduce the lag time between the surgical removal of the patient tumor sample and the orthotopic injection into the mouse brain, cells were not counted. The prepared tumor sample was directly implanted into the brains of NSG^TM^ mice (NOD-*scid* IL2Rgamma^null^, NOD-*scid* IL2Rg^null^, NOD *scid* gamma, Jackson Laboratory, Bar Harbor, ME, USA). The injection site was 2 mm right, 1 mm anterior to the bregma suture. One µL of the prepared tumor sample was injected each minute for a total of 3 min. Mice were monitored daily for the first week after surgery for recovery. Mice were then assessed 3 times per week for signs of illness associated with tumor growth. Brains were collected from mice euthanized at morbidity. Frozen tissue was stored at −80 °C, for protein or nucleic acid preparations. PFA-preserved tissue was processed and embedded in paraffin. Five micrometer sections were cut for histology and IF-FISH.

### 2.12. Statistical Analyses

Data from at least two independent experiments with individual technical replicates wherever applicable were collected. Representative images or blots are shown. Results are shown as mean ± SD. GraphPad Prism 8.0.1 was used to perform statistical analysis. One- or two-way ANOVA followed by a post hoc Dunnett’s or Tukey test, wherever applicable, was used to analyze the data.

## 3. Results

### 3.1. Presence of Both ALT and Telomerase in pHGG Patients

**Figure 1:**
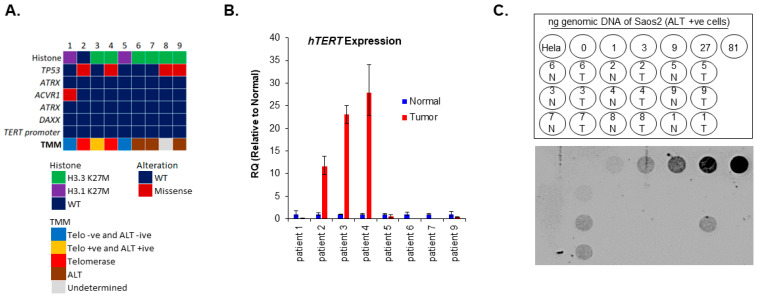
TMM in pHGG. (**A**) Graphical summary of gene mutation status and associated TMM in our DIPG cohort. Each column represents one patient. (**B**) Evaluation of *hTERT* expression by qPCR. Error bars represent the standard error of the mean from two different experiments run in triplicates. Due to a limited amount of tissue sample, patient 8 is not shown. (**C**) Dot blot of the C-Circle assay to evaluate ALT activity. Serial dilutions of Saos2 were used as a positive for ALT control and HeLa cells were used as negative control for ALT. N = normal, T = tumor.

We have previously demonstrated, in a multi-institutional retrospective study, high TERC and *hTERT* expression in 46% of non-brainstem pHGG samples as compared to non-neoplastic controls. Evidence of ALT was noted in 53% of non-brainstem pHGG specimens. ALT and telomerase use were identified in 21% of non-brainstem pHGG comparatively to other studies [11,21,26,39]. However, no association between ALT use and progression-free survival or overall survival (OS) were observed [21]. Moreover, in the HERBY trial, a recent comprehensive study in non-brainstem pHGG, *TERT* promoter mutations or amplifications were identified. However, a follow-up on TMM was not performed [40]. We evaluated the TMM in 9 DIPG patients’ derived tumors matched with normal brain tissue. ALT and telomerase activity were detected in 3/9 (33%) and 2/9 (22%) respectively. Interestingly, 1/9 (11%) of the tumors showed both ALT and telomerase activity, while 22% (2/9) of the tumors displayed no evidence of any TMMs (ALT negative and Telomerase negative) (Figure 1A–C). Furthermore, we observed that tumors utilizing ALT were all mutated in *H3-3A* (H3.3K27M) but did not harbor *ATRX* or *DAXX* mutations (Figure 1A–C). A similar presence of ALT in pHGG in the absence of *ATRX* mutations has recently been reported [26].). Taken together, our data demonstrate the presence and relevance of inter- as well as intra-tumor molecular heterogeneity of TMM in pHGG.

**Figure 2:**
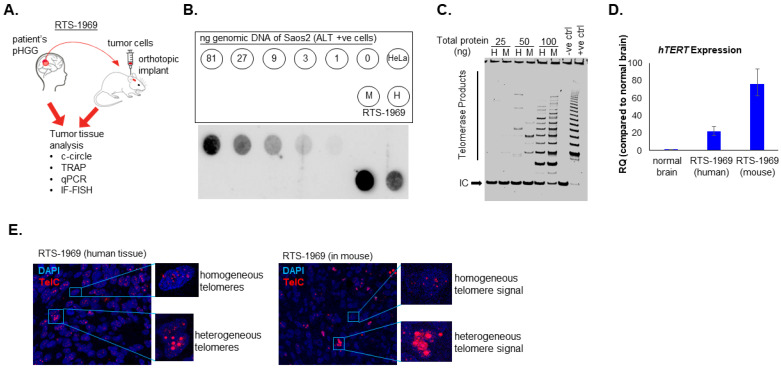
RTS-1969 tumor shows characteristics of both Telomerase and ALT phenotype. (**A**) Schematic illustration of in vivo orthotopic tumor transplant in mouse. (**B**) Dot blot of the C-Circle assay to evaluate ALT activity. Serial dilutions of Saos2 were used as a positive control for ALT and HeLa cells were used as negative control for ALT. (**C**) TRAP assay showing telomerase activity in RTS-1969 from patient tumor (H) as well as in mouse xenograft (M). (**D**) Evaluation of *hTERT* expression by qPCR. Error bars represent the standard error of the mean from two different experiments run in triplicates. (**E**) IF-FISH images to assess intensity of telomere signal in tumor and non-tumor regions of the brain from GBM patient and mouse xenograft tissue.

Given the coexistence of both ALT-positive and telomerase activity in pHGG shown here and elsewhere by our group [21], we evaluated the TMM in an orthotopic mouse model of pHGG harboring a H3.3G34R mutation (Figure 2A). We observed that both the primary patient tumor and mouse patient’s derived xenograft were ALT positive (Figure 2B). Interestingly, these tumors also showed evidence of telomerase activity detected by the TRAP assay (Figure 2C) and an increased expression of *hTERT* (Figure 2D). Furthermore, using IF-FISH, we observed cells with heterogeneous telomere signals in both human and mouse orthotopic tumors, indicative of the ALT phenotype (Figure 2E). Similarly, cells with homogenous telomeres signal, a feature of telomerase activity, were also observed (Figure 2E). Together, these data provide evidence of tumor TMM heterogeneity with the coexistence of both ALT and telomerase activity in the same tumor and this phenotype is conserved in mouse orthotopic xenograft, thereby providing a unique opportunity to concurrently test in vivo ALT and telomerase inhibitors.

### 3.2. The Sensitivity of pHGG Cells to ATR and CHK1 Inhibitors Is Not Specific to the Presence of ALT Activity

**Figure 3:**
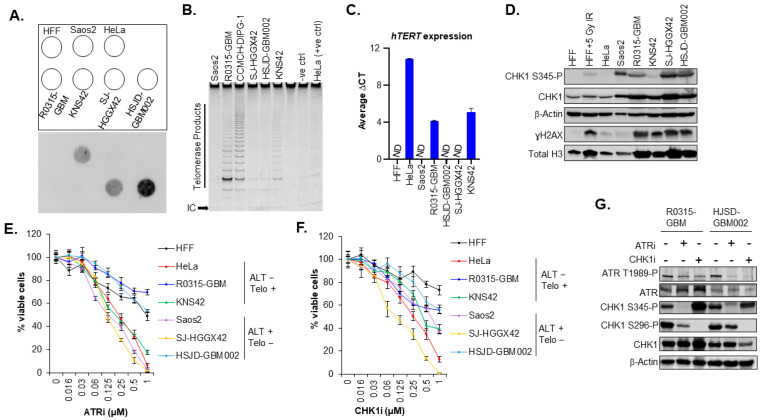
TMM in pediatric GBM cell lines and their sensitivity to ATR and CHK1 inhibitor. (**A**) Dot blot of the C-Circle assay to test for ALT activity in different pediatric cell lines. Saos2 cells were used as a positive control and HeLa cells were used as negative control for ALT. (**B**) TRAP assay showing telomerase activity in pediatric GBM cell lines. HeLa cells were used as positive control. (**C**) Evaluation of *hTERT* expression by qPCR. Error bars represent the standard error of the mean from two different experiments run in triplicates. (**D**) Western blot analysis of CHK1-S345-P (ATR phosphorylation site), CHK1-S296-P (autophosphorylation site), total CHK1, ɣH2AX, and total H3 in pediatric GBM cell lines, HeLa, Saos2, and HFF cells. β-Actin served as a loading control. Irradiated HFF cells with 5 Gy were used as a positive control for CHK1 activation cells. (**E**) Proliferation assay for ATR kinase inhibitor-treated pediatric GBM cells. Cells were treated with the indicated concentrations of ATR inhibitor for 6 days. Proliferation was determined by the WST1 assay. Error bars represent SD; experiment was performed in duplicate. ALT−, Telo+ are ALT negative and telomerase positive cell lines, while ALT+, Telo− are ALT positive and telomerase negative cell lines (**F**) proliferation assay for CHK1 inhibitor-treated pediatric GBM cells. Cells were treated with the indicated concentrations of CHK1 kinase inhibitor for 6 days. Proliferation was determined by the WST1 assay. Error bars represent SD; experiment was performed in duplicate. ALT−, Telo+ are ALT negative and telomerase positive cell lines, while ALT+, Telo− are ALT positive and telomerase negative cell lines. (**G**) Western blot analysis of ATR T1989-P, ATR, CHK1-S345-P (ATR phosphorylation site), CHK1-S296-P (autophosphorylation site), total CHK1 in our pediatric GBM cell lines treated either with ATR or CHK1 kinase inhibitor at predetermined IC_50_ for 48 h. β-Actin served as a loading control.

It has previously been shown that cancer cell lines including osteosarcoma, lung cancer and glioma stem cell lines that rely on the ALT pathway were hypersensitive to the inhibition of the protein kinase ataxia telangiectasia- and RAD3-related protein (ATR) [41]. However, later studies could not confirm a general ATR inhibitor sensitivity of ALT-positive cells [42,43]. Given these findings, we evaluated the sensitivity to ATR inhibitor and ATR downstream target CHK1 inhibitor in our panel of pediatric GBM cell lines. First, we assessed the telomerase and *hTERT* expression using TRAP assay and qPCR respectively. ALT activity was assessed using the C-Circle assay (Figure 3A–C). Two cell lines, SJ-HGG42 and HSJD-GBM002, harboring the H3.3G34R mutation were ALT positive, while KNS42 and R0315-GBM cell lines were telomerase positive and were H3.3G34V mutant and H3.3 WT, respectively (Figure 3A–C). Saos-2 cells and HeLa were used as positive controls for ALT and telomerase activity, respectively. HFF cells were used as normal diploid primary human cells and *hTERT* expression negative control. Next, we explored the correlation between the sensitivity to the inhibitors of the DNA damage pathway (ATR and CHK1 inhibitors) and ALT status in our cell lines. We first evaluated the status of the ATR pathway (Figure 3D and Figure S1A,B). Our results indicate that the sensitivity to ATR inhibition is not specific to ALT cells compared to cells using telomerase. (Figure 3E and Figure S1C). Furthermore, CHK1, a downstream target of ATR, has previously been shown to be the kinase for H3.3 S31 phosphorylation required for chromatin integrity and cell survival of ALT cells [44]. Like ATR inhibition, the sensitivity to CHK1 inhibition was not specific to ALT-positive cells (HJSD-GBM002) compared to telomerase-positive cells (Figure 3F and Figure S1C). Moreover, it has been shown that H3.3 S31 phosphorylation levels are elevated in ALT cells compared to telomerase-positive cells, and more importantly, there is an aberrant localization and spreading of H3.3 S31 phosphorylation along the chromosome arms during mitosis in ALT cells [44]. However, in our GBM cell lines, aberrant staining and localization of H3.3 S31 phosphorylation along the chromosome arms was not specific to ALT but present in both ALT-positive HSJD-GBM002 cells and telomerase-positive R0315-GBM cells (Figure S2), suggesting that H3.3 S31 phosphorylation is not specifically required for pHGG ALT cells survival. Together, these data suggest that the ATR-CHK1 pathway is not a specific determinant of ALT phenotype in pediatric GBM.

## 4. Discussion

We have shown that the ALT phenotype is enriched in DIPG tumors with H3.3K27M mutation consistent with our previous data [21]. However, in pHGG, previous studies have demonstrated a stronger incidence of ALT activation in H3.3G34R/V-mutants pHGG (100%) compared to H3.3K27M-mutant pHGG (40%) [11]. Furthermore, in our DIPG cohort, we did not observe any *ATRX/DAXX* or *TP53* mutations in our ALT tumors, which is consistent with previous findings demonstrating the presence of ALT activity without *ATRX/DAXX* alterations [11,26]. In contrast to non-DIPG pHGG where *ATRX* mutations and ALT association occur with a high frequency, *ATRX/DAXX* mutations are not relevant to the ALT pathway in DIPG. Furthermore, mutations in the *IDH1* gene [45], as well as depletion of histone chaperone ASF1 [46], have been implicated in the activation of the ALT phenotype, indicating that ALT activation in tumor cells is not solely dependent on *ATRX/H3-3A* mutations. Further investigations in a larger cohort are needed to elucidate the mechanistic biology of ALT in DIPG.

Previous reports indicated a better prognosis in ALT adult GBM, with a less aggressive phenotype and better patient survival [15,21,22,23,24,25]. However, ALT activation confers a poor prognosis in other cancers, including neuroblastoma and soft tissue sarcomas [24,47,48]. Moreover, previous studies did not find a definitive association between ALT status and OS in pHGG [21,26], except in ALT-positive and *TP53* mutant DIPG with improved OS [49]. In our DIPG cohort, one patient harbored both ALT and telomerase activation. Interestingly, two patients were telomerase and ALT negative. Together, these data indicate the TMM heterogeneity and the variability of ALT determinants in pHGG. The presence of both ALT and telomerase activity in patient tumors may be due to the documented pHGG intra-tumoral heterogeneity of pHGG [34,38,50,51]. Previous studies have demonstrated the coexistence of both telomerase and ALT pathways especially intra-tumoral heterogeneity in telomere lengths and TMM activity, with ALT and telomerase functioning in different cells within the same tumor or within the same cells in the tumor [31,32,33]. Our data indicate the presence of both ALT and telomerase activity within a GBM tumor isolated from a patient and this presence was conserved in our orthotopic mouse model that can be used for targeting both ALT and telomerase pathways. The coexistence of high *hTERT* expression and ALT was shown to significantly reduce the OS in neuroblastoma patients [31]. In contrast, it has been demonstrated that some highly malignant cancers such as melanoma and neuroblastoma can occur in the absence of any TMM because of extensive telomere reserves [52,53]. Our previous study also showed 16% of non-brainstem HGG and 18% of DIPG patients demonstrated no canonical TMM [21], in comparison to 40% seen in adult GBM [54]. Taken together, these observations emphasize the importance of TMMs in pHGG tumorigenesis and response to therapy and support the ongoing evaluation of telomere-based therapeutic interventions, including targeting telomerase and ALT activity.

The inherent requirement of tumor cells to utilize TMMs elicits clinical targeting of these pathways. Previous research and early trials have shown encouraging results for telomerase targeting [37,55,56]. However, the approach to therapeutic targeting of ALT is still elusive due to the complexity of the ALT mechanism and the heterogeneity of ALT tumors in tumorigenesis and response to treatment. Previous refuted studies stated the hypersensitivity of ALT-positive cells to ATR inhibitors [41]. However, ATR sensitivity of tumors cells is not specific to ALT [42,43], which is consistent with our findings in pediatric GBM cell lines. Although there is sensitivity to ATR inhibition, our current data and previous studies show that ATR inhibition alone may not be sufficient to target tumors with ALT activity. Furthermore, ATR downstream target, CHK1 has previously been shown to be the kinase for H3.3 S31 phosphorylation, enabling the aberrant localization and spreading of H3.3 S31 phosphorylation along the chromosomal arm of ALT cells during mitosis. This process was shown to be essential for ALT cell survival by preventing activation of the DNA damage response and apoptosis during mitosis [44]. However, in our pHGG cells, both CHK1 inhibition and aberrant spread of H3.3 S31 phosphorylation on the chromosome arms were not specific to ALT cells but also observed in telomerase-positive cells, suggesting that CHK1 kinase activity and H3.3 S31 phosphorylation are not required for pHGG ALT cell survival. Notwithstanding, exploitation of the genetic instability and DNA damage response often observed in ALT cells could be essential for targeting these tumors. Indeed, previous studies indicated the interference of the ALT pathway with DNA damage response, thereby providing radiation resistance to glioma stem cells [57]. Further studies on potential combination therapies, including ATR inhibition and radiation therapy, may improve the targeting of ALT tumors.

## 5. Conclusions

Our study demonstrated the TMM heterogeneity in pHGG tumors. With 44–53% of non-brain stem pHGG and over 20% of DIPG using ALT as a TMM, elucidating the molecular pathway of ALT is a prerequisite for the development of new therapeutic strategies to improve outcomes in this vulnerable group of patients.

## Data Availability

The data presented in this study are contained within this article.

## Supplementary Materials

The following supporting information can be downloaded at: https://www.mdpi.com/article/10.3390/cancers15123070/s1, Figure S1. Sensitivity of pediatric GBM cell lines to ATR and CHK1 kinase inhibitors (A,B). Bar graphs showing the quantification of CHK1-S345-P, and ɣH2AX levels from western blot data (Figure 3D). OD (optical density) was measured by densitometry analysis using ImageJ software. Band intensities were normalized to total CHK1 or total H3 as applicable. C. IC_50_ values for our pediatric GBM cell line treated either with ATRi or CHK1i. (D–F). Bar graphs showing the quantification of ATR T1989-P, CHK1-S345-P and CHK1-S296-P from western blot data (Figure 3G). OD (optical density) was measured by densitometry analysis using ImageJ software. Band intensities were normalized to total ATR, CHK1 as applicable. Figure S2. H3.3 S31 phosphorylation spread across the chromosome is not specific to ALT. Representative IF staining for H3.3 S31-P (green) in asynchronous HSJD-GBM002 and R0315-GBM cells. H3 S10-P is used as M-phase marker (red), and the nuclei were stained with DAPI (blue). Figure S3. Uncropped immunoblots for Western blot figures. Compilation of uncropped immunoblots for all Western blot figures. The positions of the molecular weight marker [kDa] are shown on the left. The uncropped immunoblots images are related to: (A) Figure 3D, (B) Figure 3G.
