# Supplementary material for: Alternative Lengthening of Telomeres in Pediatric High-Grade Glioma and Therapeutic Implications
Source: Cancers (Basel). 2023 Jun 6;15(12):3070. doi: 10.3390/cancers15123070 (PMC10296514; doi:10.3390/cancers15123070)

Umrau, supplementary Figure S1

A.

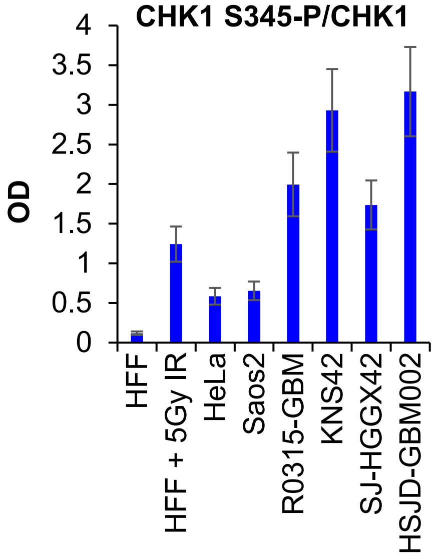

B.

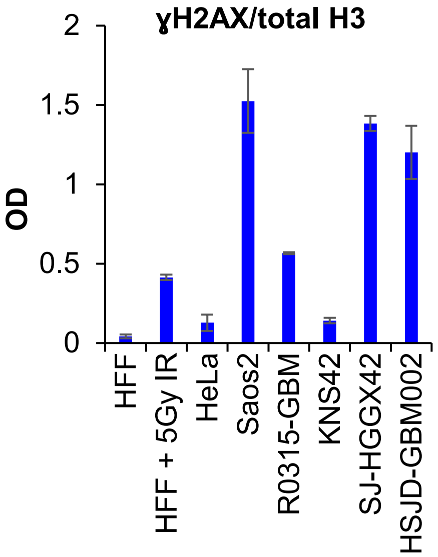

C.

	IC$_{50}$ (μM)	
**Cell lines**	**ATRi**	**CHK1i**
HFF	4.17	2.08
HeLa	0.48	0.24
Saos2	1.00	0.50
R0315-GBM	1.25	0.62
KNS42	1.00	0.50
SJ-HGGX42	0.22	0.11
HSJD-GBM002	2.09	1.05

D.

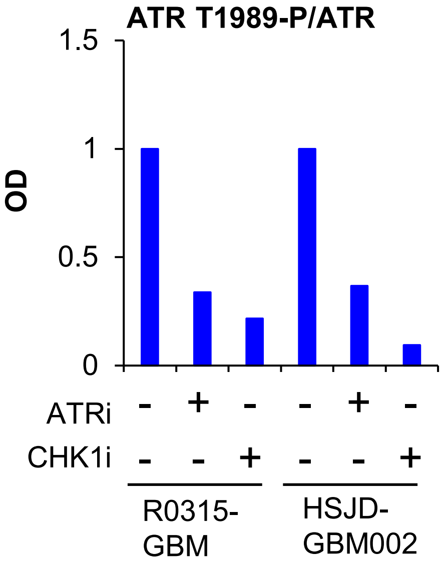

E.

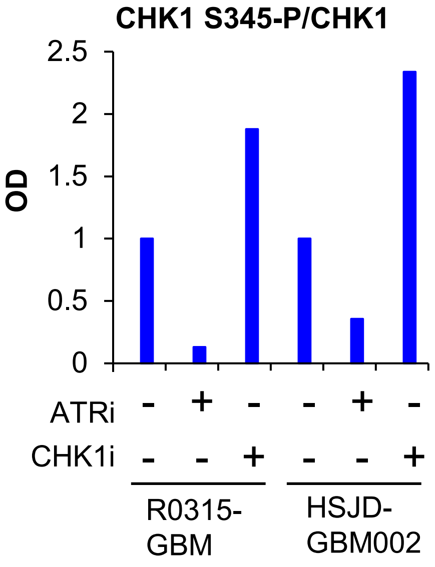

F.

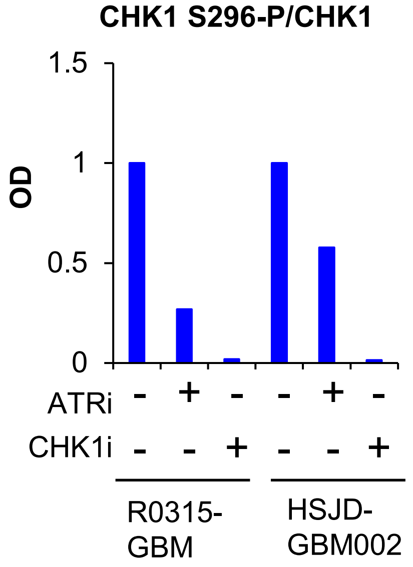

Umrau, supplementary Figure S2

HSJD-GBM002

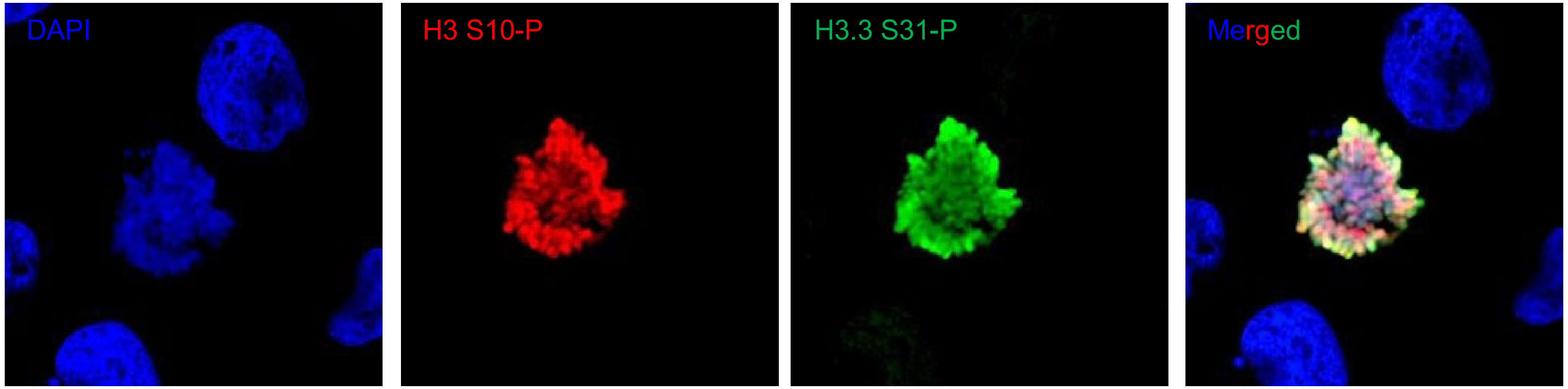

R0315-GBM

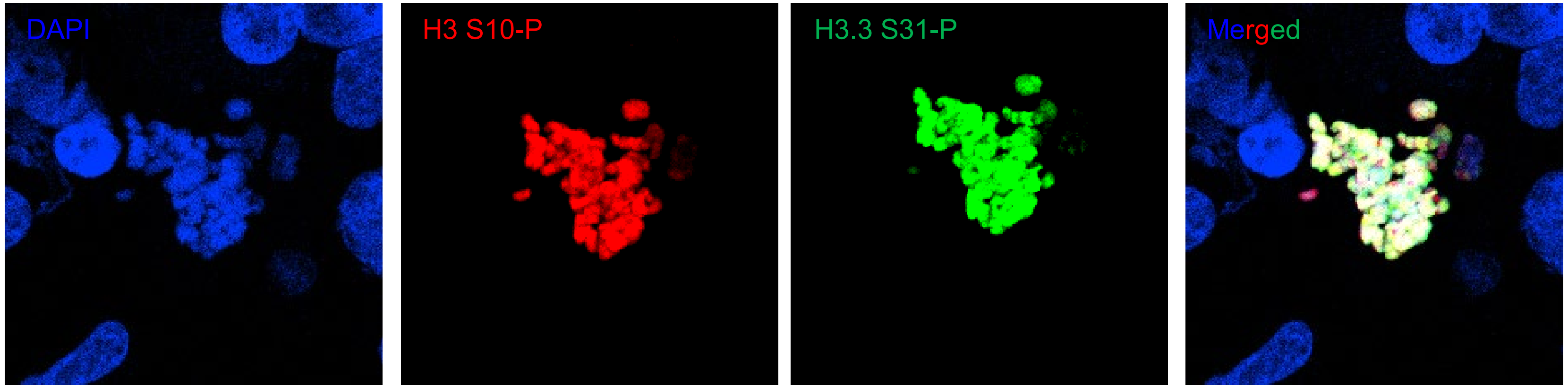

Umaru, supplementary Figure S3A

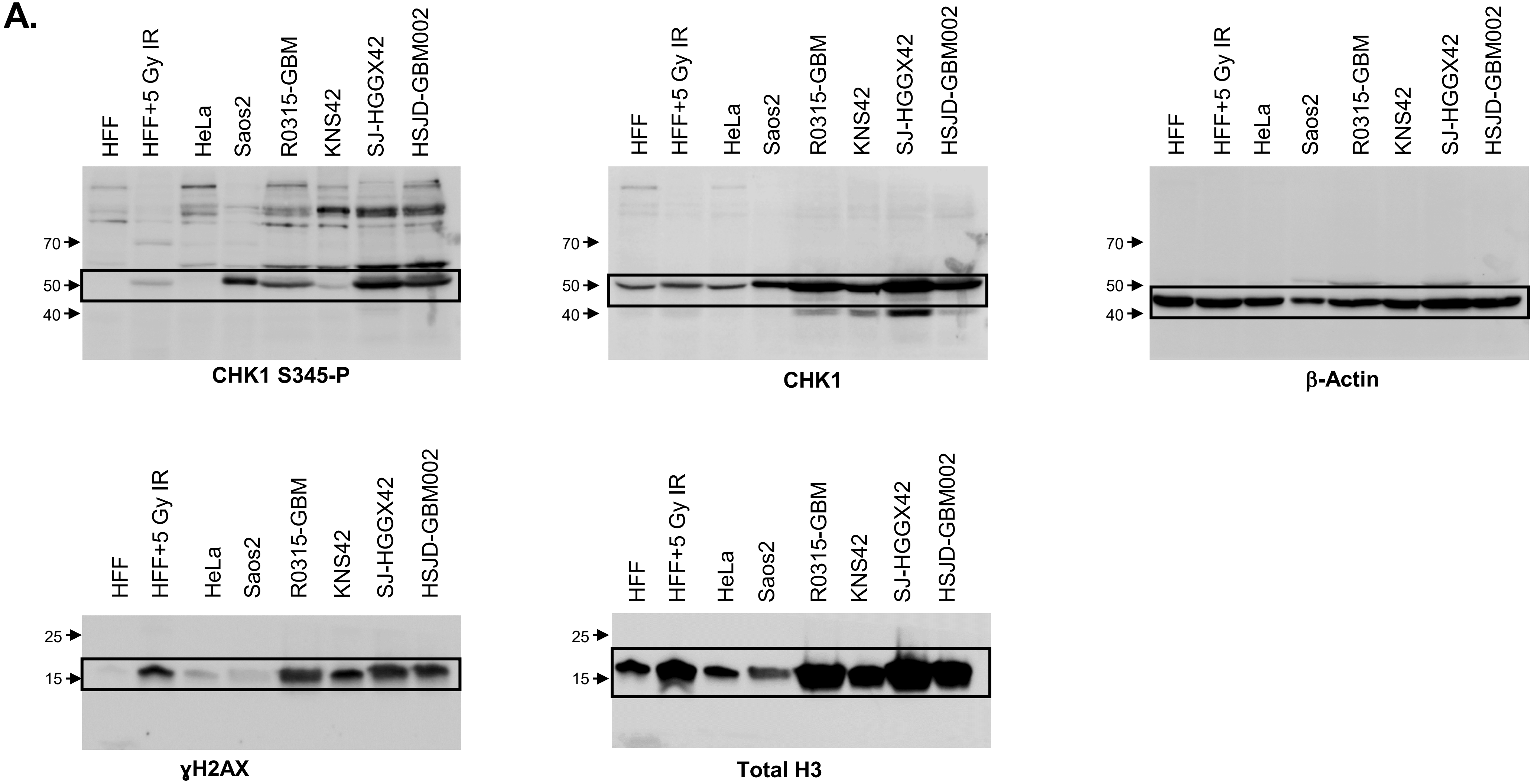

Umaru, supplementary Figure S3B

B.

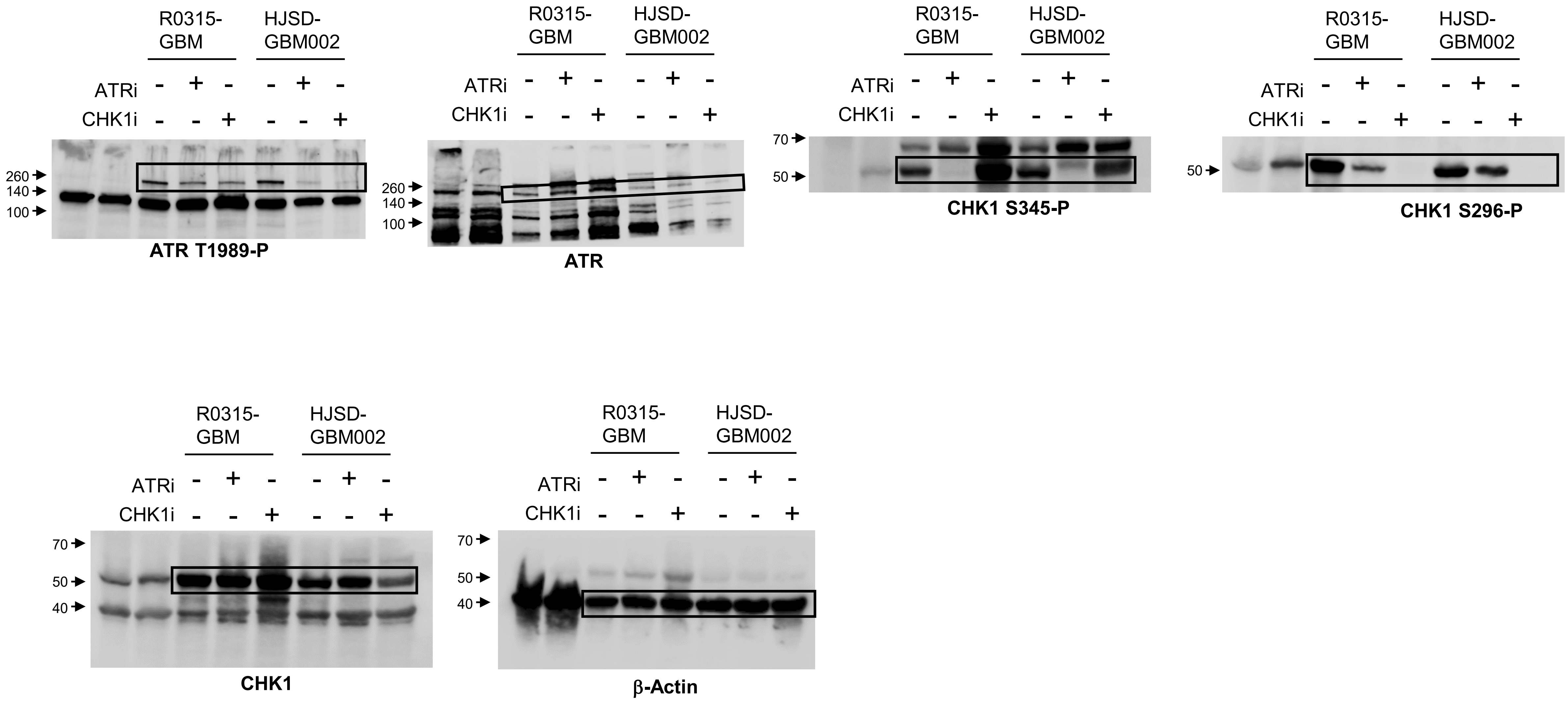

Supplement: Supplementary file 1 [file cancers-15-03070-s001.zip › cancers-2412810-supplementary.pdf]
